# A High Sensitivity AlN-Based MEMS Hydrophone for Pipeline Leak Monitoring

**DOI:** 10.3390/mi14030654

**Published:** 2023-03-14

**Authors:** Baoyu Zhi, Zhipeng Wu, Caihui Chen, Minkan Chen, Xiaoxia Ding, Liang Lou

**Affiliations:** 1 School of Microelectronics, Shanghai University, Shanghai 201800, China; 2 The Shanghai Industrial μ Technology Research Institute, Shanghai 201899, China

**Keywords:** MEMS hydrophone, PMUT, leak detection, leak localization

## Abstract

In this work, a miniaturized, low-cost, low-power and high-sensitivity AlN-based micro-electro-mechanical system (MEMS) hydrophone is proposed for monitoring water pipeline leaks. The proposed MEMS Hydrophone consists of a piezoelectric micromachined ultrasonic transducer (PMUT) array, an acoustic matching layer and a pre-amplifier amplifier circuit. The array has 4 (2 × 2) PMUT elements with a first-order resonant frequency of 41.58 kHz. Due to impedance matching of the acoustic matching layer and the 40 dB gain of the pre-amplifier amplifier circuit, the packaged MEMS Hydrophone has a high sound pressure sensitivity of −170 ± 2 dB (re: 1 V/μPa). The performance with respect to detecting pipeline leaks and locating leak points is demonstrated on a 31 m stainless leaking pipeline platform. The standard deviation (STD) of the hydroacoustic signal and Monitoring Index Efficiency (MIE) are extracted as features of the pipeline leak. A random forest model is trained for accurately classifying the leak and no-leak cases using the above features, and the accuracy of the model is about 97.69%. The cross-correlation method is used to locate the leak point, and the localization relative error is about 10.84% for a small leak of 12 L/min.

## 1. Introduction

Seventy-five percent of the earth’s surface is covered with water. However, only 0.75% of freshwater resources can be used directly. At this stage, water for urban residents is mainly supplied through water distribution networks (WDNs). WDNs can become broken and leak due to unreasonable pipeline design, pipeline material problems, corrosion, aging, construction quality and other human factors and natural factors [1]. R. Liemberger et al. [2] stated that the global non-revenue water (NRW) is at 126 billion cubic meters each year, and the total NRW accounts for 30% of the water supply. The breakage of WDNs not only causes a waste of freshwater resources and economic losses but also pollutes water resources and endangers the health of residents.

The two main purposes of monitoring pipeline leaks are leak detection and leak localization. The common monitoring methods can be classified as acoustic detection methods and non-acoustic detection methods [1,3,4,5,6,7,8]. Non-acoustic detection methods include specific district-metered areas (DMAs), gas injection, ground penetrating radar, chemical tracing, etc. Acoustic detection methods consist of artificial listening, accelerometer sensing, fiber optic detection, hydrophones, noise loggers, etc. Compared with non-acoustic detection methods, acoustic detection methods have better application prospects due to their low cost and high suitability for large-scale deployment [6]. Among these methods, the hydrophone is intrusively mounted on the pipeline and directly contacts with the water to monitor the hydroacoustic signal in the pipeline. Since hydroacoustic signals are less affected by the environment outside the pipeline and low-frequency hydroacoustic signals possess small attenuation, hydrophones can monitor a leak at a longer distance and have high location accuracy [9,10,11,12,13,14]. Piezoceramic-based hydrophones have been successfully applied to pipeline leak monitoring, especially in the monitoring of plastic pipelines with high hydroacoustic signals attenuation [14,15,16,17,18]. However, their high cost and large size limit their large-scale deployment [3].

With the development of MEMS technology, MEMS-based sensors, including MEMS accelerometers and MEMS hydrophones, are being applied to pipeline leak monitoring [19]. Ismail et al. compared the performance of a number of commercial MEMS accelerometers on plastic pipelines in terms of the number of axes, sensitivity, price and power consumption [20]. They found that a commercial MEMS accelerometer with a higher number of axes had higher accuracy. The reason was that the X-axis of the commercial MEMS accelerometer could not identify the pipeline condition, while the other two axes could. Tariq et al. trained four machine learning models (KNN, Decision, Random Forest and AdaBoost) to detect pipeline leaks using the signals captured using a MEMS accelerometer [21]. They verified that Random Forest was the algorithm with the highest accuracy. Xu et al. proposed a low-cost, tiny MEMS hydrophone sensor for water pipeline leak detection [22]. The MEMS hydrophone device was packaged with a customized onboard preamplification circuit using an acoustic matching material. The MEMS hydrophone was able to capture the leak signal and locate its position. Phua et al. proposed a smart hydrophone sensing network combining MEMS hydrophones and the Internet of Things (IoT) [23]. By analyzing the transient signals captured by the MEMS hydrophone, critical leak acoustic information could be obtained. The location of the leak point was calculated by the cross-correlation method for different distances and leak rates. However, MEMS hydrophones still have some problems in terms of pipeline leak detection and location accuracy and their performance needs to be improved.

The types of MEMS hydrophones can be divided into those based on capacitive micromachined ultrasonic transducers (CMUTs) and those based on piezoelectric micromachined ultrasonic transducers (PMUTs). CMUTs have high sensitivity, but the gap between the capacitive poles is small, their fabrication is difficult and a high DC bias voltage needs to be added for operation [24]. However, PMUTs do not require high DC bias voltage for low-power operation and have high sensitivity and good linear response [25]. Due to these advantages, PMUTs have been widely used in fluid density monitoring [26,27,28,29,30], ultrasonic positioning [31], intravascular ultrasound (IVUS) [32] fingerprint sensors [33] and hydroacoustic monitoring. Among the studies for hydroacoustic monitoring, Xu et al. proposed a hydrophone based on a PMUT with a resonant frequency of 1.086 MHz, and the sensitivity of the hydrophone was measured to be −182.5 dB [34]. Jia et al. proposed a hydrophone based on a honeycomb architecture PMUT with a resonant frequency of 0.96 MHz, and the sensitivity of the hydrophone measured to be −178 dB [35]. However, the sensitivity of these MEMS hydrophones does not meet the needs of long-distance pipeline monitoring. Thus, there is a need to develop high-sensitivity MEMS hydrophones.

In this work, we design a high-sensitivity MEMS hydrophone for pipeline monitoring. The MEMS hydrophone consists of a PMUT, an acoustic matching layer and a pre-amplifier amplifier circuit. The first-order resonant frequency of the PMUT is 41.58 kHz. The proposed MEMS hydrophone has a sensitivity of −170 ± 2 dB (re: 1 V/μPa) after optimizing the acoustic matching layer and pre-amplifier amplifier circuit. The MEMS hydrophone installed on a 31 m stainless pipeline platform is able to monitor both leak and non-leak acoustic signals. For monitoring water pipeline leaks, a Random Forest model is trained using STD and MIE of the hydrophone signal, which can achieve an accuracy of 97.69%. After filtering by a high-pass filter, the hydrophone signal is used to locate the leak point based on a cross-correlation algorithm, and the localization relative error is as low as 10.84% for a small leak of 12 L/min.

## 2. The Design of the MEMS Hydrophone

### 2.1. The Working Principle and Fabrication of PMUT

**Figure 1:**
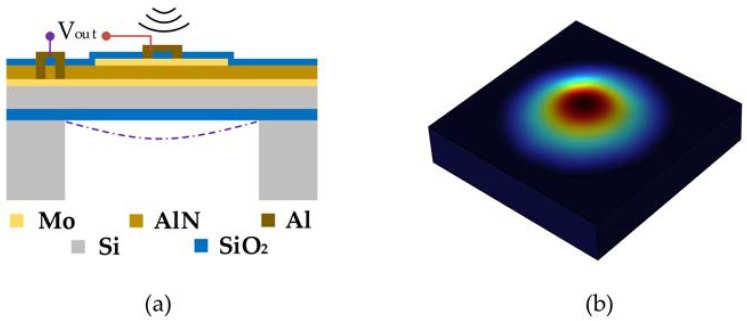
The PMUT structure: (**a**) cross-sectional view, (**b**) simulated mode shape of a sensing diaphragm in the PMUT.

The proposed MEMS hydrophone is based on the PMUT which works through the piezoelectric effect of the piezoelectric material. The hydroacoustic signals cause mechanical deformation of the piezoelectric film and produce transverse stress, which generates induced charges at the electrode and realizes the conversion between the acoustic and electrical domains, as shown in Figure 1a. The performance of the PMUT has a direct effect on the hydrophone. The performance of the PMUT is related to the material of the piezoelectric layer [24]. The sensitivity of the hydrophone is proportional to the *e*_31*f*_/*ε* of the piezoelectric material [34,35,36], and the commonly used piezoelectric materials in PMUTs include the lead zirconium titanate (PZT), zinc oxide (ZnO) and aluminum nitride (AlN), whose *e*_31*f*_/*ε* are −0.0083, −0.231 and −0.093 respectively. AlN is chosen for the hydrophone in this study because it has good reception characteristics and is compatible with CMOS technology.

As is shown in Figure 1a, the stacked structure of the PMUT consists of, from bottom to top, a cavity, a silicon oxide layer, a silicon device layer, a lower electrode Mo layer, a piezoelectric layer AlN layer, an upper electrode Mo layer and a SiO_2_ protection layer. The corresponding geometric parameters of the PMUT are summarized in Table 1. The resonant frequency of the PMUT in water is calculated according to Equation (1) [37],

(1) $$f_{\mathrm{water}}=\frac{f_{\mathrm{air}}}{\sqrt{1+\frac{0.67d\rho_{\mathrm{water}}}{\rho_{\mathrm{eq}}}}}$$

(2) $$f_{\mathrm{air}}=\frac{1}{2\pi }\sqrt{\frac{k}{m}}=\frac{\lambda_{0}\times t}{2\pi \times d^{2}}\sqrt{\frac{E_{\mathrm{eq}}}{12\times \rho_{\mathrm{eq}}\times (1-\upsilon_{\mathrm{eq}}^{2})}}$$

where *d* is the diameter of the diaphragm, *t* is the thickness of the diaphragm, *E*_eq_ is the equivalent elastic modulus, *ρ*_eq_ is the equivalent density, *ν*_eq_ is the equivalent Poisson’s ratio and *λ*_0_ is the correction factor. The first-mode shape of a single sensing diaphragm of the PMUT is simulated in COMSOL Multiphysics, as shown in Figure 1b. And the simulated frequency is 41.80 kHz.

**Figure 2:**
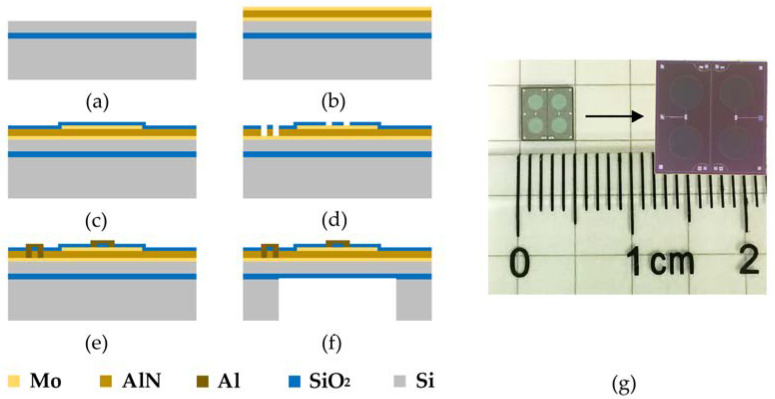
The PMUT array and MEMS hydrophone: (**a**–**f**) process flow, (**g**) optical image of the PMUT.

The PMUT is fabricated on the silicon-on-insulator (SOI) platform of the Shanghai Industrial µ Technology Research Institute (SITRI). Its fabrication process is shown in Figure 2a–f [38,39]. Figure 2a shows the customized single-sided polished SOI wafer. As shown in Figure 2b, the Mo bottom electrode layer, AlN piezoelectric layer and Mo top electrode layer are deposited on this SOI wafer, in that order. After the top electrode is patterned, a protective layer of SiO_2_ is deposited to prevent oxidation, as shown in Figure 2c. Then, in order to pattern the pad positions, the SiO_2_ protective layer is patterned by Reactive Ion Etching (RIE) and the AlN piezoelectric layer is patterned by etching, as shown in Figure 2d. AlN pads are deposited to collect the charge from the top and bottom electrodes, as shown in Figure 2e. Figure 2f shows that the back cavity is released by Deep Reactive Ion Etching (DRIE). The fabricated PMUT is a 2 × 2 array with a size of 5 mm × 5 mm as is shown in Figure 2g.

### 2.2. Characterizations of PMUT

**Figure 3:**
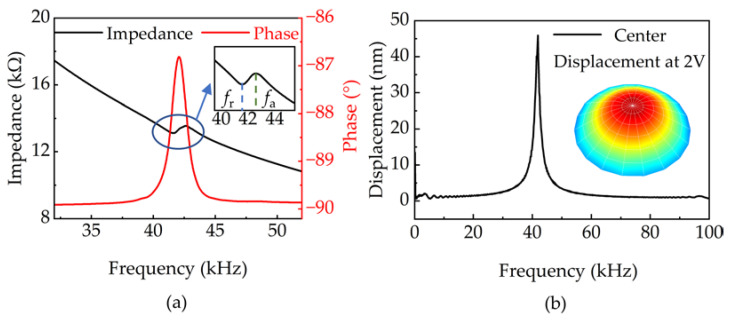
Characterizations of PMUT: (**a**) impedance and phase curves of the PMUT, (**b**) frequency response measurement results using LDV.

The fabricated PMUT was characterized, and the results are shown in Figure 3. The electrical parameters of the PMUT were characterized by an impedance analyzer (KEYSIGHT E4799A). Figure 3a indicates that the resonant frequency (fr) is 41.58 kHz and the anti-resonant frequency (fa) is 42.60 kHz. The phase is −86.7° at 42.10 kHz. According to the test results of the impedance curve, the effective electromechanical coupling coefficient *K_eff_* of the PMUT is calculated as 2.42% according to Equation (3),

(3) $$K_{eff}^{2}=1-{\left(\frac{f_{r}}{f_{a}}\right)}^{2}$$

The frequency response of PMUT was measured through an LDV (Polytec UHT-120) under a sinusoidal frequency sweep (100 Hz–100 kHz, 2 Vpp). As shown in Figure 3b, the resonant frequency was measured to be 41.88 kHz. The displacement at the first-order resonant frequency is 46 nm. The 1st mode shape of the diaphragm at the resonant frequency is also shown in Figure 3b.

### 2.3. Package and Characterizations of MEMS Hydrophone

**Figure 4:**
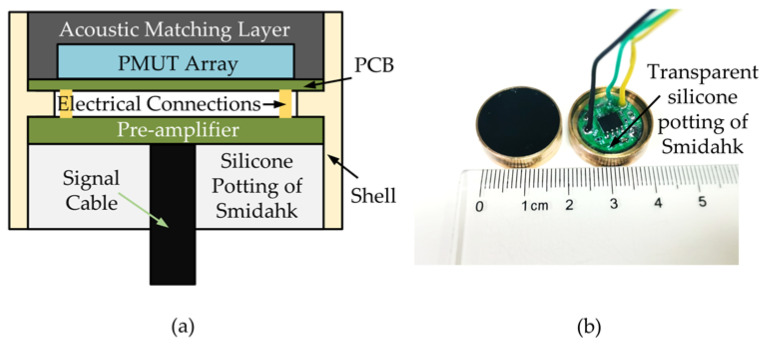
MEMS hydrophone assembling and packaging: (**a**) the structure of the hydrophone, (**b**) photograph of the packaged hydrophone.

The MEMS hydrophone is packaged with a PMUT, an acoustic matching adhesive, a pre-amplifier amplifier circuit and silicone Smidahk sealant. The structure of the hydrophone is shown in Figure 4a. Firstly, the PMUT is attached to the PCB for electrical connection. Then the PCB is mounted to the shell and the surface of the PMUT is encapsulated with an acoustic matching layer. Finally, the pre-amplifier amplifier circuit is electrically connected to the PCB. The pre-amplifier amplifier circuit is packaged with silicone Smidahk sealant. The packaged hydrophone is a 20 mm diameter cylinder, as shown in Figure 4b. The acoustic matching layer is mainly used to match the impedance of the water and reduce the energy loss caused by the reflection of the acoustic signal. The transmission coefficient of sound [35] at the interface of the two media can be expressed as Equation (4),

(4) $$T=\frac{4Z_{1}Z_{2}}{{\left(Z_{2}+Z_{1}\right)}^{2}}$$

where *Z*_1_ and *Z*_2_ are the acoustic impedance of the two media of propagation. For the proposed hydrophone, *Z*_1_ is the water, and *Z*_2_ is the acoustic matching adhesive. The acoustic impedance of water *Z* is about 1.57 MRayl. In order to achieve acoustic impedance matching, the acoustic impedance should be as close to it as possible. The acoustic matching layer is polyurethane, which has an acoustic impedance of 1.50 MRayl. Due to the mass loading of the acoustic matching adhesive, the resonant frequency of the MEMS hydrophone is measured to be 10.67 kHz. The function of the pre-amplifier circuit is to amplify the signal and achieve impedance matching. The applied amplifier (OPA827) has a noise voltage density of 4 nV/√Hz (at 1 kHz) and a noise current density of 2.2 fA/√Hz (at 1 kHz). It has a gain of 40 dB and a flat response from 10 Hz to 10 kHz. The silicone Smidahk sealant is resistant to moisture and aging, and it can protect the pre-amplifier circuit for a long time in water.

**Figure 5:**
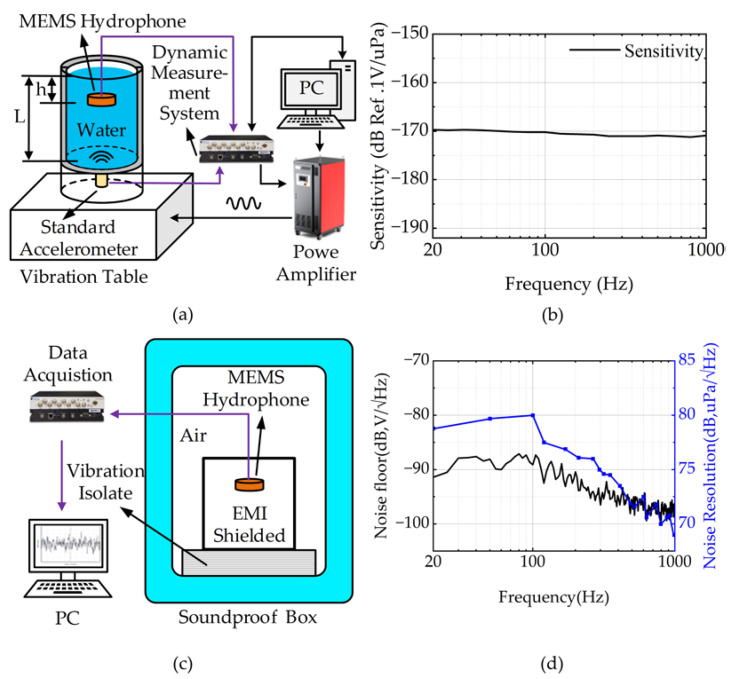
MEMS Hydrophone characterization and results: (**a**) vibrating liquid column calibration setup, (**b**) the receiving sensitivity of the MEMS hydrophone, (**c**) schematic diagram of the noise calibration device, (**d**) the noise density and noise resolution of the MEMS hydrophone.

The packaged MEMS hydrophone was tested using a vibrating table and the sensitivity of the hydrophone was calibrated through the vibrating liquid column method. As shown in Figure 5a, the calibration system includes a vibrating table, a standard accelerometer and a dynamic test system (Spider-80x). The procedure for testing the sensitivity of a MEMS hydrophone with a shaker is as follows. First, the hydrophone is installed at a distance h from the liquid level. Then, the PC is used to control the dynamic acquisition system and to set the frequency and acceleration of the vibration signal for the test. The vibration signal is output to the shaker through the power amplifier. At the same time, the dynamic acquisition system monitors in real-time to ensure the correct magnitude and frequency of acceleration of the shaker through standard accelerometer signals, thus forming a closed-loop vibration system. Finally, the dynamic acquisition system collects the electrical signal of the MEMS hydrophone. The sensitivity of the hydrophone [40] is calculated by Equation (5),

(5) $$S_{M}=\frac{U_{M}}{U_{a}}\times \frac{S_{a}}{\rho_{h}}$$

where *U_M_* and *U_a_* are the output voltages of the MEMS hydrophone and a standard accelerometer, respectively. *S_a_* is the sensitivity of the standard accelerometer. *h* is the distance between the MEMS hydrophone and the liquid level and ρ is the density of the liquid. As shown in Figure 5b, the sensitivity curve of the MEMS hydrophone is relatively flat. This shows that the proposed MEMS hydrophone has a bandwidth of 20 Hz to 1000 Hz with a sensitivity of −170 ± 2 dB (re: 1 V/μPa).

The noise density of the MEMS hydrophone was measured using a noise calibration device. As shown in Figure 5c, the noise calibration is conducted in a soundproof box to isolate the environmental interference. An electromagnetic shielding (EMI) box is also placed on the vibration isolator of the soundproof box, which can isolate electromagnetic interference from the external environment. The noise density of the MEMS hydrophone was measured to be −91 dB at 100 Hz, and the noise resolution was measured to be 79 dB at 100 Hz (re:1 upa/√Hz), as shown in Figure 5d.

## 3. Results and Discussion

### 3.1. Experimental Setup

**Figure 6:**
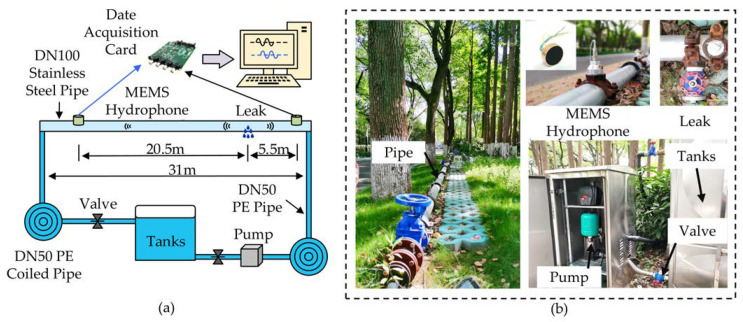
Details of the test pipeline system: (**a**) schematic diagram of the test pipeline system, (**b**) real picture of the test pipeline system.

As shown in Figure 6, the pipeline leakage test platform was also built at SITRI. It is a circulating water system composed of a 31 m DN100 stainless steel pipeline, two sound DN50 PE coils for the attenuation pump and return valve, a water tank, pump and valves. The schematic diagram of the test platform is shown in Figure 6a. Two MEMS hydrophones are installed 26 m apart at both ends of the pipeline. At a distance of 5.5 m from the MEMS hydrophone is a 7 mm diameter circular hole, which is used to simulate leakage. The hydroacoustic signals sensed by the two MEMS hydrophones are collected simultaneously through the MCC data acquisition card (USB-2020) with a sampling frequency of 20 kHz, and the collected signals are transmitted to the PC for processing.

A photograph of the actual test platform is shown in Figure 6b. A 31 m stainless steel pipeline is fixed at 10 cm from the ground and the MEMS hydrophone is intrusively installed in the pipeline to monitor the hydroacoustic signal in the pipeline. A valve is installed outside the leak point to control the size of the leak by adjusting the degree of opening of the valve. A 2 m^3^ water tank can provide the required water for the leak experiment continuously for a short period of time by means of a circulating water supply, and the water pressure in the pipeline can be changed by adjusting the degree of opening of the return valve.

### 3.2. The Characteristics of Pipeline Acoustics

**Figure 7:**
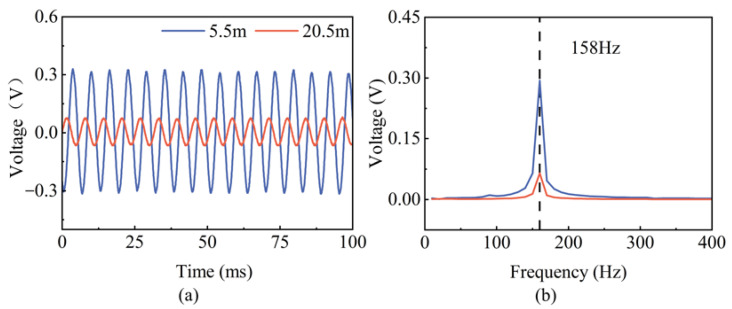
Time domain and normalized frequency domain plots of a 158 Hz acoustic signal: (**a**) Time domain plot of the two hydrophones, (**b**) Normalized frequency domain plot of the two hydrophones.

Without imposing high water pressure, an underwater loudspeaker is placed at the location of the pipeline leak to simulate a sound source with a fixed frequency of 158 Hz. The signal generated by the underwater loudspeaker is received by the MEMS hydrophones at both ends. The time domain plot and frequency domain plot of the 158 Hz acoustic signal collected by the two MEMS hydrophones are shown in Figure 7. As can be seen, the MEMS hydrophones installed on the pipe can correctly receive the acoustic signal inside the pipe without drifting at that frequency. Through analyzing the time domain signal, it is obvious that there is a certain attenuation and delay of the signal because of the different distances of acoustic signal propagation between the source and the two hydrophones. The attenuation of the sound signal at 158 Hz is 1.9 dB/m in this experimental pipe.

### 3.3. The Characteristics of Leakage Sound

**Figure 8:**
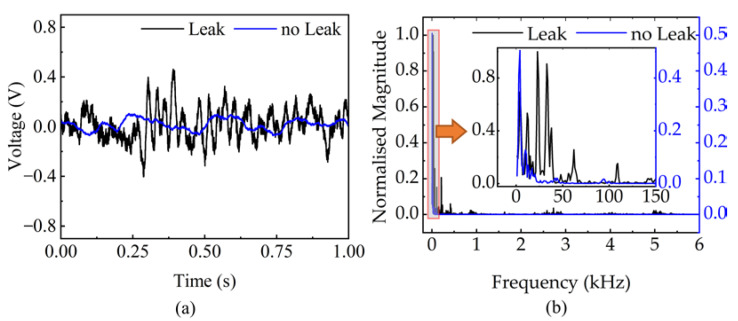
Leak and non-leak acoustic signal analysis: (**a**) time-domain measurement results of no leak and leak, (**b**) normalized frequency domain measurement results of no leak and leak.

The characteristics of the acoustic signals captured by the hydrophones are relevant to subsequent leak detection and location. The non-leak signal is collected from one of the MEMS hydrophones mounted at the end of the pipe (far-end MEMS hydrophone) at a water pressure of 2.5 bar. Whereas the leak signal is collected from one of the MEMS hydrophones mounted at the end of the pipe at a constant water pressure of 2.5 bar and a leak volume of 24 L/min. As shown in the time domain diagram of the signal in Figure 8a, there is a significant difference between the amplitude of the leak and non-leak signals. The statistical values of the leakage and non-leakage signals are given in Table 2. Figure 8b is the normalized frequency response after performing an FFT on the signal, which shows that the non-leak signals are mainly distributed below 50 Hz. The non-leak signal is an external noise, which is mainly due to interference coming from the pump and valve.

### 3.4. Leak Detection

A leak detection algorithm based on the STD and MIE was developed since there is a significant difference in the amplitude of the signals collected by the hydrophone in the leak and non-leak conditions. The STD and MIE are used as features for supervised training of the Random Forest model. The Random Forest algorithm is a Bagging integration algorithm composed of multiple decision trees, which can reduce the possibility of error in the judgment of a single model. Its parameters are set as follows: Number of trees = 100; criterion= gini; maximal depth = 10; minimum number of samples of leaf nodes = 10; minimum number of samples = 160.

**Figure 9:**
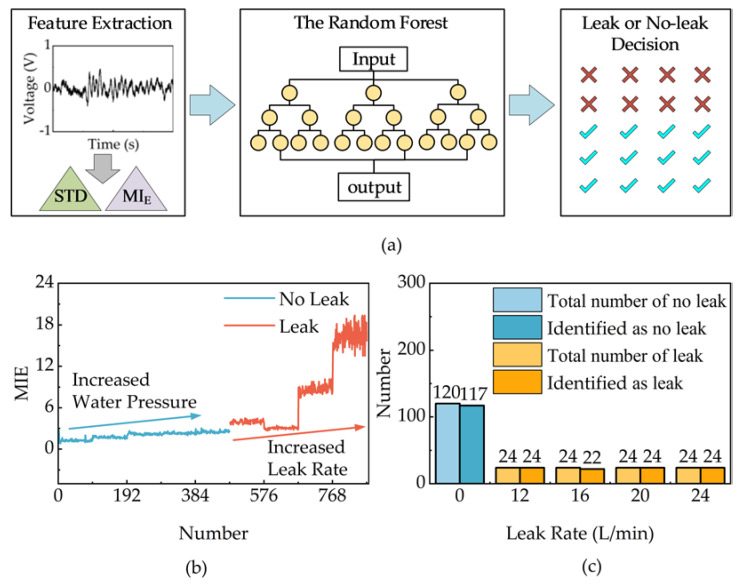
Leak detection chart: (**a**) the specific implementation process, (**b**) MIE value of the training dataset after filtering, (**c**) the results of the validation dataset after filtering.

In the non-leak condition, the far-end MEMS hydrophone collected signals for 10 min at water pressures of 2 bar, 2.5 bar, 3 bar, 3.5 bar and 4 bar respectively. In the leak (at a leak pressure of 2.5 bar) condition, the far-end MEMS hydrophone collected signals for 10 min at leak rates of 12 L/min, 16 L/min and 20 L/min respectively. Based on the previous analysis, the leak signal is pre-processed with high-pass 50 Hz filtering to reduce the interference of noise signals. As shown in Figure 9a, the specific implementation process of the leak detection algorithm is as follows:

1. STD is calculated every 5 s. The non-leak signals return 600 STD values, and the leak signals return 480 STD values.
2. The monitoring threshold *MI*_0_ is established. *MI*_0_ is the average of the ten smallest STDs in the non-leak and leak cases. It is obtained by Equation (6) [21],
  (6) $$MI_{0}=mean(\sigma_{j},10)$$
3. Calculating the *MIE*. The non-leak signals return 600 *MIE* values, and the leak signals return 480 *MIE* values. The *MIE* is obtained through Equation (7) [21],
  (7) $$MIE=\frac{\sigma_{j}}{MI_{0}}$$
4. Training the Random Forest model. The STD and MIE are used as features for supervised training. Before the application of the Random Forest, the data are separated randomly according to an 8:2 ratio, of which 80% are used to train the Random Forest model and 20% are used to validate the model.
5. Validating the model. The accuracy is obtained through Equation (8) [21],
  (8) $$accuracy=\frac{TP+TN}{TP+FN+FP+TN}$$
  where true positive (*TP*) means a positive event identified correctly, true negative (*TN*) means a negative event identified correctly, false negative (*FN*) means a positive event incorrectly identified as negative and false positive (*FP*) means a negative event incorrectly identified as positive.

The MIE values of the training dataset after filtering are shown in Figure 9b. There is a clear difference between the MIE values in the leak and non-leak cases. In the non-leak case, the MIE increases as the pressure in the pipe increases. In the leak case, the difference in MIE values between a leak rate of 12 L/min and 16 L/min is small, and the difference between a leak rate of 20 L/min and 24 L/min is large. The final validation result is shown in Figure 9c. Among the 200 sets of non-leak verification data, only 3 sets are incorrectly judged as leaking. In the leak verification data, two sets of data are incorrectly judged as non-leaking in the leak rate of 16 L/min. The result means that the accuracy of the proposed method is 97.69%.

### 3.5. Leak Localization

**Figure 10:**
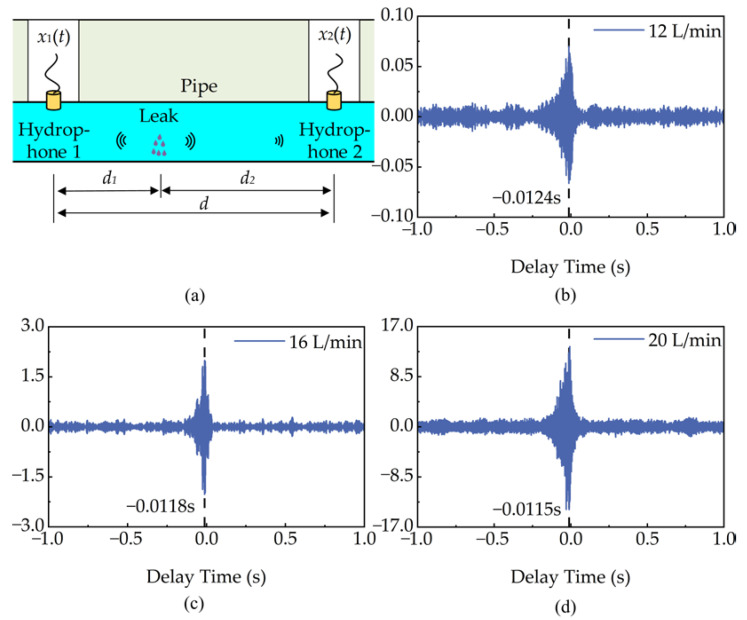
The cross-correlation localization: (**a**) Schematic diagram showing MEMS hydrophones deployment for pipeline leak detection, (**b**) leak delay of 12 L/min, (**c**) leak delay of 16 L/min, (**d**) leak delay of 20 L/min.

As shown in Figure 10a, the principle of the cross-correlation localization is as follows: two MEMS hydrophones are placed on both sides of the pipeline water leakage point; the two MEMS hydrophones receive the water leakage sound signal and generate two continuously varying electrical signals *x*_1_(*t*) and *x*_2_(*t*); the MEMS hydrophones are placed at different distances, the time difference in the propagation of the leaking water sound can be calculated by the cross-correlation method. The distance of hydrophone 1 from the leakage point can be obtained through Equation (9),

(9) $$d_{1}=\frac{d-c\tau }{2}$$

where *d* indicates the distance between the two hydrophones, *τ* indicates the delay time, *c* is the speed of sound propagation in the liquid-filled pipeline [41], and its expression is shown below,

(10) $$c=c_{f}{\left(1+\frac{2B_{f}a}{E_{p}h}\right)}^{-1/2}$$

where *B_f_* is the bulk modulus of the fluid and *a* is the radius of the pipeline. *E_p_* represents the Young’s modulus of the pipeline. *h* represents the wall thickness of the pipeline. *c_f_* is the wave velocity of the fluid and *τ* is calculated by the cross-correlation function. The cross-correlation function is defined as follows [41],

(11) $$R_{x_{1}x_{2}}(\tau )=E\left[x_{1}(t)x_{2}(t+\tau )\right]$$

where *E[]* is the mathematical expectation.

The leak rate is set to be 12 L/min, 16 L/min and 20 L/min, respectively and the leak point is located through the cross-correlation. In order to improve the positioning accuracy and reduce the interference of low-frequency noise below 50 Hz, a high-pass filter is applied. As shown in Figure 10b–d, the delay times of the cross-correlation are −0.0124 s, −0.0118 s and −0.0115 s, respectively. The localization relative error is calculated using Equation (12). The calculated localization distance and localization relative error are shown in Table 3. It can be seen that the localization relative error decreases with increasing leak rate and the 20 L/min leak rate has the best positioning accuracy. In addition, the 12 L/min leakage rate has the highest localization relative error of 10.84%. Since low-energy hydroacoustic signals are easily interfered with by background noise at a small leakage rate, the localization relative error at this time is higher than that of a large leakage rate.

(12) $$\delta =\frac{\Delta }{L}\times 100\%$$

where Δ is the absolute error and *L* is the real value.

## 4. Conclusions

In this work, a high-sensitivity MEMS hydrophone with a sensitivity of −170 dB is proposed for pipeline monitoring and shows a significant improvement in sensitivity compared to hydrophones used for leak monitoring reported in the literature [22,23]. A 31 m pipeline leak monitoring platform is built to demonstrate the performance of the proposed MEMS hydrophone. A Random Forest model algorithm for leak detection is developed. The accuracy of leak detection is up to 97.69%. A basic cross-correlation method for locating leak points is also developed. The localization relative error with this method is as low as 10.84% at a small leakage rate of 12 L/min. The feasibility of the proposed MEMS hydrophone for leak monitoring is initially demonstrated on the pipeline leak test platform. However, the environment of the actual underground pipeline is more severe. In our next step, the proposed MEMS hydrophone will be applied to actual pipelines to verify its performance.

## Figures and Tables

**Table 1:** Geometric parameters of the PMUT.

Material	Top Mo	AlN	Bottom Mo	Si	Cavity
Radius (µm)	690	-	-	-	950
Thickness (µm)	0.2	1	0.2	5	400

**Table 2:** The statistical values of the leakage and non-leakage signals.

	Mean Value	STD	Maximum Value	Minimum Value
Leakage	0.01608	0.12466	0.46154	−0.40293
Non-leakage	0.01833	0.04378	0.10501	−0.07082

**Table 3:** Localization distance and localization relative error.

Leak Rate	Delay Time	Distance	Relative Error
12 L/min	−0.0124 s	4.904 m	10.84%
16 L/min	−0.0118 s	5.303 m	3.58%
20 L/min	−0.0115 s	5.502 m	0.04%

## Data Availability

Not applicable.
